# Transcriptome dataset of mouse adipose tissue across estrous cycles

**DOI:** 10.1038/s41597-024-03942-5

**Published:** 2024-10-05

**Authors:** Hongjie Zheng, Yier Bai, Shan Wu, Zhixuan Jiang, Qing Pei, Min Yao

**Affiliations:** 1 Department of Plastic and Reconstructive Surgery, Shanghai Ninth People’s Hospital, Shanghai Jiao Tong University School of Medicine, 639 Zhizaoju Road, Shanghai, 200011 China; 2 Department of Burn, Ruijin Hospital, Shanghai Jiao Tong University School of Medicine, 197 Ruijin 2nd Road, Shanghai, 200025 China

**Keywords:** RNA sequencing, Genetics research

## Abstract

Adipose tissue is crucial for energy storage and release, ensuring energy homeostasis within the body. Disturbances in the physiology of adipose tissue have been associated with various health disorders, such as obesity and diabetes. The reproductive cycle represents a fundamental biological pattern in female physiology. Although previous research has highlighted the substantial regulatory influence of ovarian hormones on adipose tissue, our understanding of the comprehensive changes in adipose tissue throughout the reproductive cycle remains limited. In this study, we examined the transcriptomic profile of female mouse-adipose tissue across their complete estrous cycles. The findings provided detailed descriptions of the datasets generated, including information on data collection, processing, and quality control. The study also demonstrated the robustness of these data through various validation steps. These findings serve as crucial resources for investigating the role of estrous cycle rhythmicity in important adipose tissue processes in the future.

## Background & Summary

Adipose tissue plays a pivotal role in human physiology, functioning as an energy reservoir and endocrine tissue^1,2^. Dysregulation of adipose tissue functions, including adipogenesis, lipolysis, adipocytokines secretion, lipid metabolism, and thermogenesis, can result in a spectrum of pathological conditions such as metabolic disorders, obesity, type 2 diabetes, cardiovascular diseases, lipodystrophy, precocious puberty, among others^1–10^. As the prevalence of these adipose tissue-related disorders continues to increase, there is a growing global interest in adipose tissue research^2^.

The reproductive cycle is a fundamental biological rhythm in female mammals; it is referred to as the menstrual cycle in women and higher-order primates and as the estrous cycle in non-primates^11–14^. During the reproductive cycle, female hormones like estrogen and progesterone undergo regular fluctuations that are associated with numerous metabolic activities^13,15–19^. Adipose tissue is widely recognized as a hormonally regulated tissue, and the regulatory effects of numerous individual hormones, such as insulin, estrogen, progesterone, testosterone, and thyroid hormones, on adipose tissue across multiple facets have been studied extensively^20–28^. However, our understanding of how adipose tissue responds within the intricate, multi-hormonal physiological dynamics of the entire reproductive cycle remains limited.

**Fig. 1:**
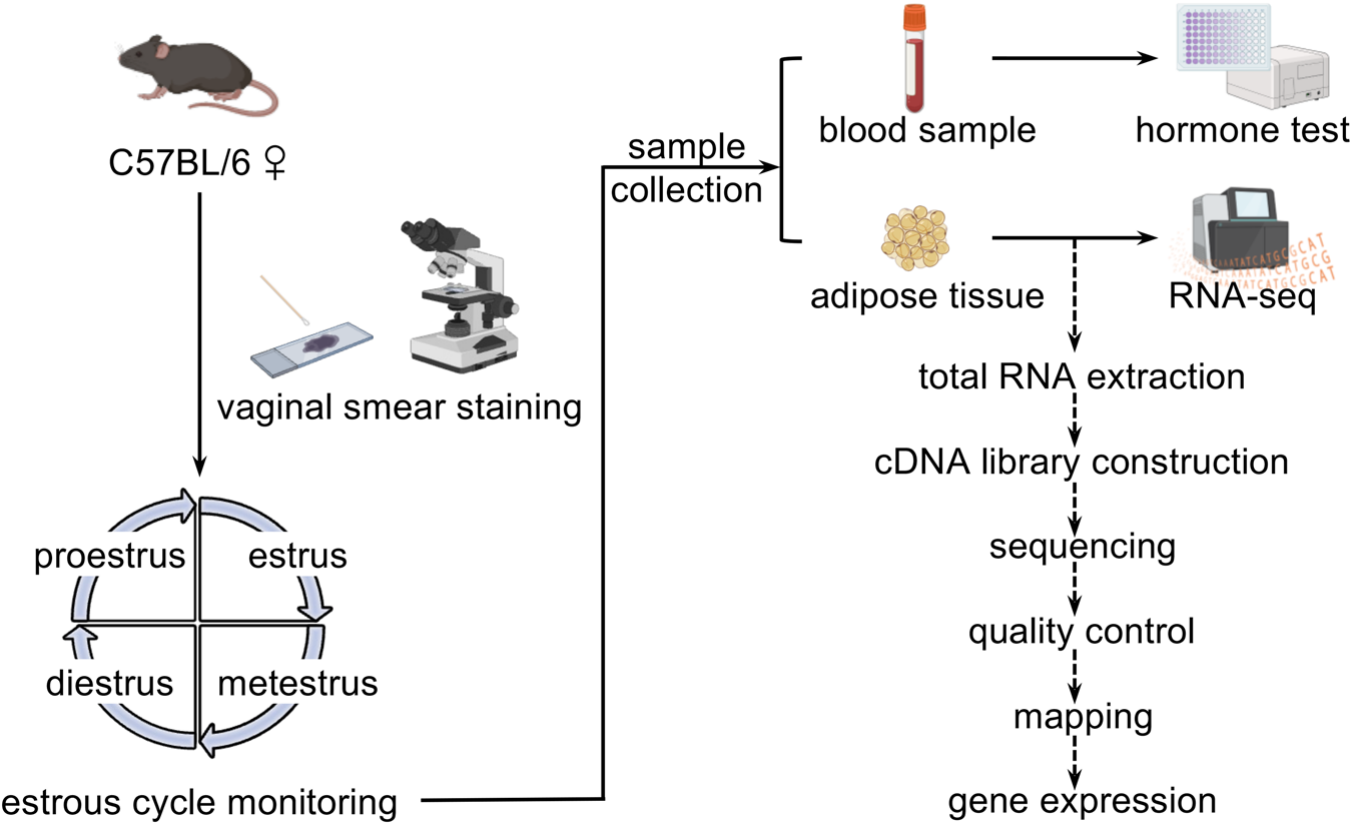
Schematic representation of the study design and data processing. Twenty 8-week-old female C57BL/6 mice were selected, with five mice allocated per stage. The estrous cycle of each mouse was monitored continuously using vaginal smear staining. Mice with consecutive estrous cycles covering the proestrus, estrus, metestrus, and diestrus were specifically chosen for subsequent experimentation. Blood samples were analyzed using enzyme-linked immunosorbent assays (ELISA) to quantify estrogen and progesterone serum levels. Adipose tissue was harvested and subjected to RNA-seq analysis using the Illumina NovaSeq 6000 platform. The figure was created using BioRender (https://www.biorender.com/).

Transcriptional information is essential for elucidating the phenotype and functionality of adipose tissue. A previous study revealed the transcriptional variances in adipose tissue between estrus and diestrus; however, information regarding the other two stages of the estrus cycle is still lacking^29^. Therefore, in this study, we conducted a comprehensive transcriptomic analysis of adipose tissue across the entire estrous cycle in mice, including proestrus, estrus, metestrus, and diestrus (Fig. 1). Twenty RNA-seq datasets were obtained. These datasets provide valuable resources for studying cyclical variations in adipose tissue across the reproductive cycle, thereby aiding in uncovering the potential mechanisms underlying metabolic disorders and optimizing therapeutic interventions to leverage cyclic variations.

## Methods

### Animals and sample collection

The experimental manipulation of the animals was approved by the Animal Care and Experiment Committee of Shanghai Ninth People’s Hospital, Shanghai Jiao Tong University School of Medicine (approval number: SH9H-2024-A1002-1). Twenty 8-week-old female C57BL/6 mice, weighing 20–25 g, were used in this study (Table S1, see Supplementary Information document). Mice were maintained in a standard laboratory environment with a temperature of 22 °C, humidity at 40–60%, and a 12-hour light-dark cycle, with unrestricted access to food and water .

**Fig. 2:**
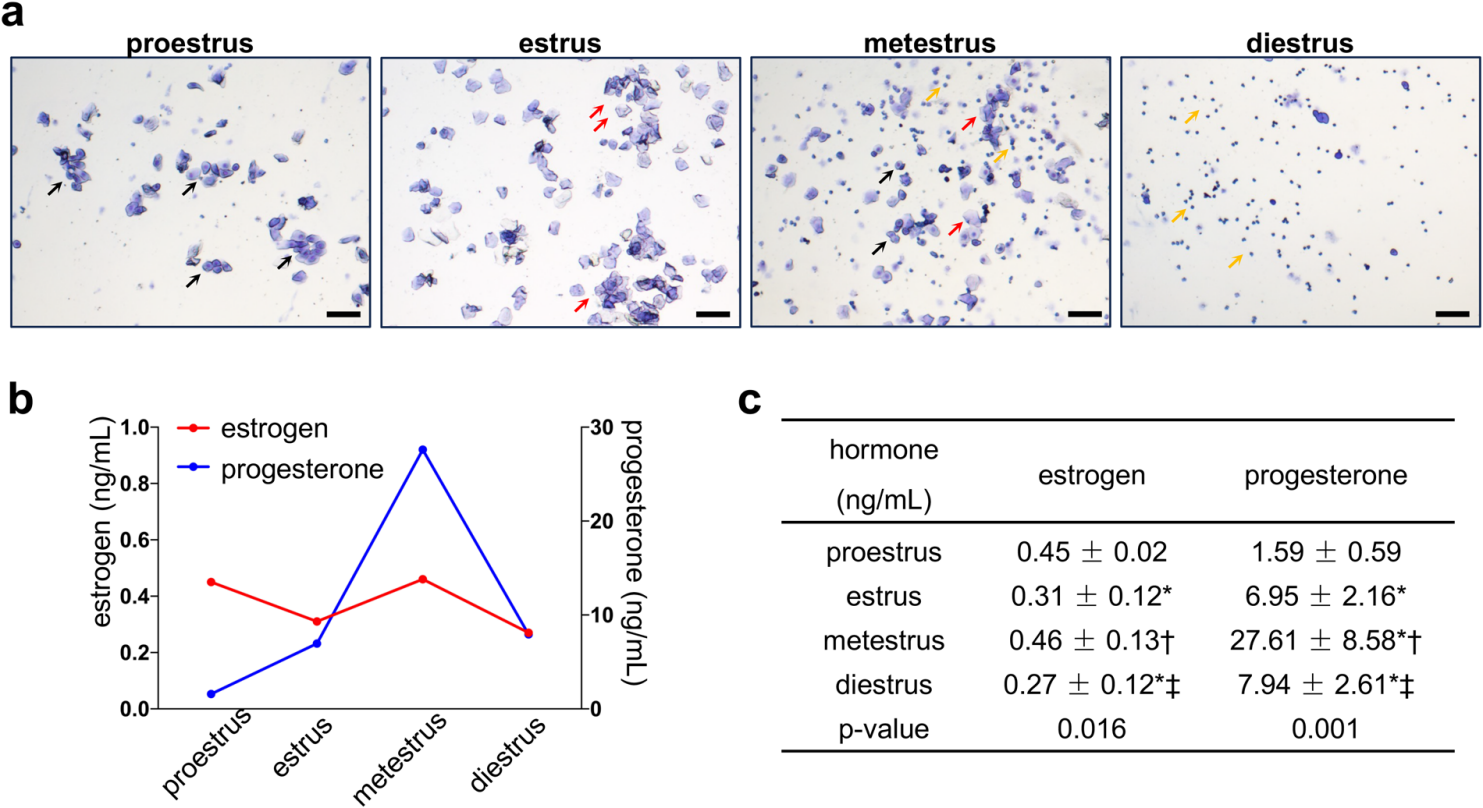
Mouse estrous cycle validation. (**a**) Vaginal smears depicting stages of the estrous cycle. Black arrows denote nucleated epithelial cells, red arrows indicate cornified epithelial cells, and yellow arrows represent leukocytes. Scale bars = 100 ${\rm{\mu }}$ m. (**b**,**c**) Ovarian hormone concentrations during the estrous cycle. Values are presented as means ± SD. *Statistically different from proestrus; †statistically different from estrus; ‡ statistically different from metestrus.

After one week of acclimatization, vaginal smears were collected from each mouse daily between 8:00 AM and 9:00 AM to confirm their estrous cycles^11,15^. Briefly, the mice were restrained and then subjected to vaginal smears by gently inserting and rolling a cotton-tipped swab wetted with 0.9% saline into the vagina. The cells collected from the swab were immediately placed onto a dry glass slide and air-dried for 10 min. Subsequently, the slides were stained with crystal violet (Beyotime, Shanghai, China) for 10 min, rinsed with water, and subsequently photographed under a light microscope (Nikon, Tokyo, Japan). The stage of the estrous cycle was microscopically identified by analyzing the types and proportions of vaginal cells as previously described^11,15^. Specifically, proestrus phase smears primarily contain nucleated epithelial cells; estrus phase smears exhibit numerous cornified epithelial cells; metestrus phase smears comprise a mixture of nucleated and cornified epithelial cells, and leukocytes in similar proportions; and diestrus phase smears are predominantly composed of leukocytes (Fig. 2a). Vaginal smears were conducted for a minimum of two cycles, and mice with consecutive estrous cycles were selected for further experimentation.

We measured serum concentrations of estrogen and progesterone to determine the stage of the estrous cycle. On the day of sacrifice, following confirmation of the estrous cycle stage via vaginal smear, the mice were anesthetized. Blood samples were then collected and centrifuged at 1000 g at 4 °C for 20 min. The resulting supernatant was stored at −80 °C for subsequent analysis of serum 17β-estradiol and progesterone levels using ELISA (Westang, Shanghai, China) (Table S1, see Supplementary Information document). Differences in hormone levels among groups were evaluated using one-way analysis of variance (ANOVA) with post-hoc Tukey’s HSD tests for pairwise comparisons in SPSS software (SPSS Statistics 29). Statistical significance was defined as an adjusted p-value less than 0.05. Estrogen levels are higher during proestrus and metestrus, and lower during estrus and diestrus (Fig. 2b,c). Progesterone levels begin to increase from proestrus, peak during metestrus, and then decline (Fig. 2b,c). Both vaginal smears and hormone levels validated the accuracy of our estrous cycle determinations.

After confirming the estrous cycle and collecting blood samples, the inguinal subcutaneous fat was harvested from the mice. Specifically, the midline of the abdomen was incised longitudinally, the skin was bluntly separated from the intermuscular space, and the inguinal fat pads were exposed and isolated. Adipose tissue samples were immediately placed in liquid nitrogen and transferred to a −80 °C freezer for storage until analysis. Cervical dislocation was performed to euthanize the mice after sampling. All samples were sent on dry ice to Shanghai Xuran Biotech (Shanghai, China) for RNA extraction, library construction, and sequencing.

### RNA sequencing

Total RNA was extracted from 20 adipose tissue samples using TRIzol reagent (Invitrogen, California, USA) as per the manufacturer’s protocol. RNA integrity was evaluated using agarose gel electrophoresis, and only high-quality RNA (28S:18S ≥ 1.5) was used for cDNA library construction (Figure S1, see Supplementary Information document). Specifically, the mRNA was first enriched using magnetic oligo (dT) beads (Thermo Fisher Scientific, Massachusetts, USA) and fragmented. Subsequently, mRNA was used to synthesize cDNA using the VVAHTS® Universal V8 RNA-seq Library Prep Kit for Illumina (NR605-0, Vazyme, China), and cDNA was purified using AMPure XP beads (Beckman Coulter, California, USA) and enriched using PCR for 15 cycles to generate the final cDNA library. Finally, sequencing was performed on the Illumina NovaSeq 6000 platform (Illumina, California, USA) with the 150 bp paired-end sequencing strategy.

### Quality control and gene expression level analysis

Twenty RNA-seq datasets of mouse adipose tissue, from proestrus to diestrus, were obtained, including five biological replicates per stage. These datasets contained a combined total of 168.86 gigabases (Gb) of raw data, averaging 8.44 Gb per sample (Table 1). We employed Skewer software (v0.2.2) to remove splice fragments and low-quality sequences from the 3′ end of the sequencing data. Following the filtering of reads based on quality and length, we retained a total of 163.57 Gb of high-quality data, averaging 8.18 Gb per sample (Table 1). Quality control analysis of the preprocessed data was conducted using FastQC (v0.11.5). Subsequently, high-quality clean reads were aligned to the reference genome (Genome Reference Consortium Mouse Build 38, GRCm38) using STAR (v2.5.3a), achieving an average mapping ratio of 94.03% (Table 1). HTSeq (v0.11.1) was employed to determine gene expression levels, generating read counts for each gene across all samples. The fragments per kilobase of transcript per million fragments mapped (FPKM) metric was used to quantify gene expression levels. A total of 35841 mRNAs were expressed (FPKM > 0) in fat samples, with variations in mRNA quantities observed across the four stages. Correlation analysis was conducted to assess the reproducibility of the biological replicates. Principal component analysis (PCA) was conducted to reduce the dimensionality of the data and the results were visualized through two-dimensional scatter plots using RStudio (v 2023.06.2 Build 561).

**Table 1:** Overview of RNA-seq data.

Sample	Raw reads	Clean reads	Raw bases (Gb)	Clean bases (Gb)	Q20% (%)	Q30% (%)	GC% (%)	Mapped ratio (%)
proestrus-1	55242920	54365052	8.34	8.04	96.70	92.20	46.50	93
proestrus-2	52220598	51527204	7.89	7.62	97.25	93.25	46.50	95
proestrus-3	50118318	49604020	7.57	7.35	97.10	92.90	47	94.10
proestrus-4	60038348	59084784	9.07	8.78	96.85	92.50	46	93.60
proestrus-5	62171092	61377900	9.39	9.08	97.25	93.10	46	94.80
estrus-1	63542826	62634704	9.59	9.24	96.85	92.70	46.50	93.50
estrus-2	52149774	51579276	7.87	7.62	97.35	93.45	46	94.50
estrus-3	63974910	63335692	9.66	9.40	97.15	93	46	94
estrus-4	58790668	58107392	8.88	8.59	97.10	92.95	46	94
estrus-5	57979766	57172442	8.75	8.45	97	92.90	46	93.20
metestrus-1	50748074	50202410	7.66	7.45	97.30	93.25	47	94.30
metestrus-2	51494806	50959258	7.78	7.56	97.40	93.40	47	94.80
metestrus-3	55467816	54834284	8.38	8.14	97.10	93	46.50	93.80
metestrus-4	51855352	51330770	7.83	7.60	97.25	93.25	46.50	93.60
metestrus-5	52201666	51537622	7.88	7.63	97.10	93	47	94.10
diestrus-1	55652992	54924730	8.40	8.13	96.95	92.80	46.50	93
diestrus-2	64557018	63885482	9.75	9.45	97.45	93.55	47	95.20
diestrus-3	52476110	51895810	7.92	7.69	97.30	93.30	46.50	94.30
diestrus-4	55336078	54680738	8.36	8.08	97.15	93.10	46	93.80
diestrus-5	52249884	51706976	7.89	7.68	97.20	93.25	46	93.90

## Data Records

The RNA-seq raw data reported in this paper have been deposited in the Genome Sequence Archive (Genomics, Proteomics & Bioinformatics 2021) in National Genomics Data Center (Nucleic Acids Res 2022), China National Center for Bioinformation/Beijing Institute of Genomics, Chinese Academy of Sciences (GSA: CRA014866) that are publicly accessible at https://ngdc.cncb.ac.cn/gsa^30^. The FPKM data from the RNA-seq have been deposited in NCBI’s Gene Expression Omnibus and are accessible through GEO Series accession number GSE275383 (https://identifiers.org/geo:GSE275383)^31^. Releases are made under the CC0 license (https://creativecommons.org/licenses/by/4.0/).

## Technical Validation

### Sequencing quality control

**Fig. 3:**
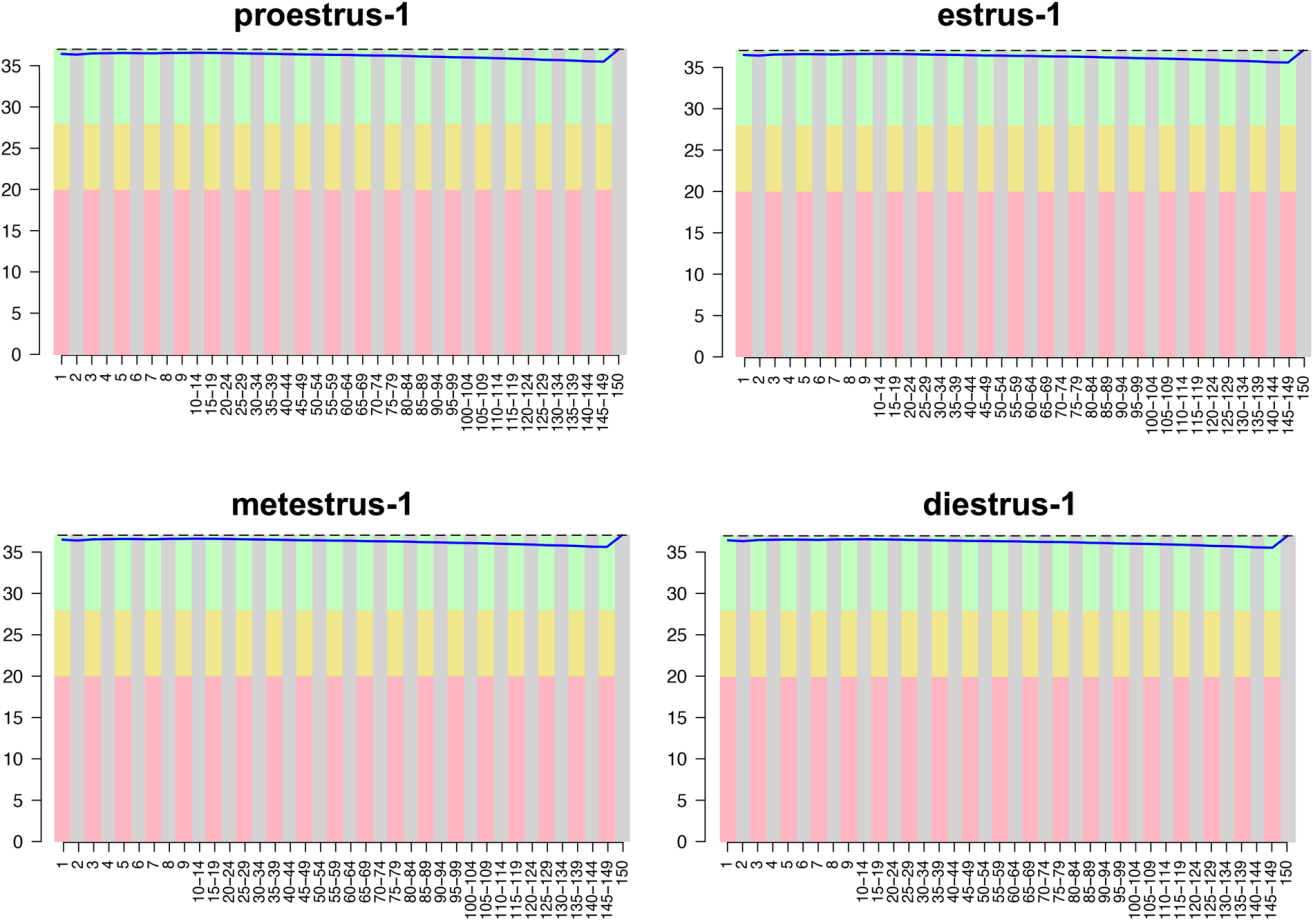
Sequencing quality score plots. The x-axis indicates the base position in the sequence, while the y-axis represents the corresponding quality scores.

Sequencing read quality was determined using FastQC, and the base ratios of Q20, Q30, and GC were calculated (Table 1). The average Q20% and Q30% values were 97.14% and 93.04%, respectively. The GC% ranged between 46% to 47% in all samples (Table 1). Moreover, the mean quality scores for each read position in every mRNA sample surpassed 30, indicating high quality (Fig. 3, Figure S2, see Supplementary Information document). These findings suggest that the sequencing data meet the requisite quality standards for subsequent analyses.

### Reproducibility assessment

**Fig. 4:**
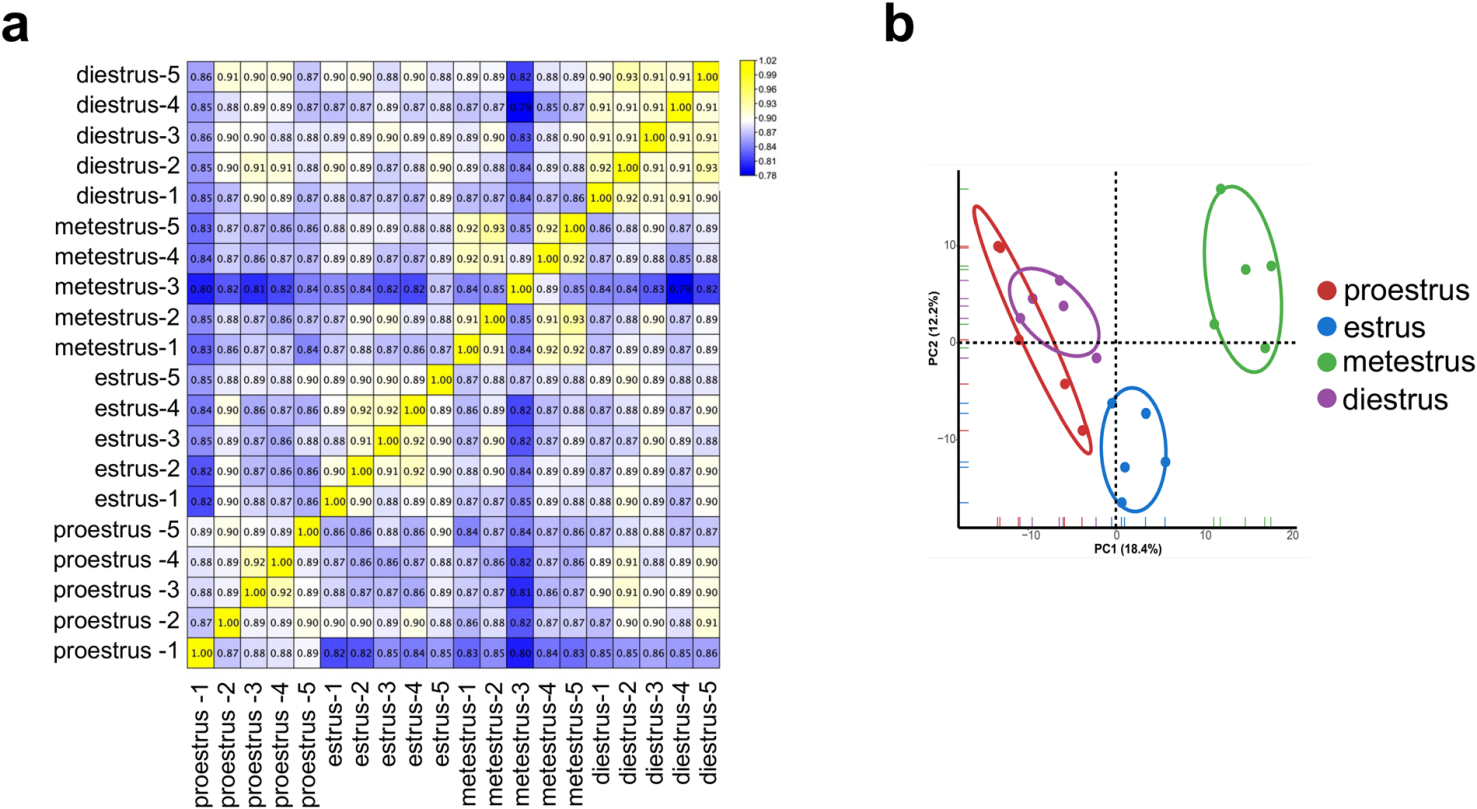
Assessment of reproducibility across biological replicates (n = 5/stage). (**a**) Correlation heatmap of 20 adipose tissue samples. (**b**) PCA of 20 adipose tissue samples.

To assess the reproducibility of the biological replicates, correlation analysis and PCA were performed. The correlation heatmap revealed higher correlation coefficients among the majority of biological replicates (Fig. 4a). Additionally, PCA illustrated that most biological replicates clustered together, while distinct separations were observed between each pair of stages (Fig. 4b). These findings strongly support the validity and reliability of our study’s data.

## Supplementary information

Supplementary Information

## Data Availability

This study employed entirely open-source software, and no custom code was developed to analyze the presented data.
