# Supplementary material for: Transcriptome dataset of mouse adipose tissue across estrous cycles
Source: Sci Data. 2024 Oct 5;11:1090. doi: 10.1038/s41597-024-03942-5 (PMC11455834; doi:10.1038/s41597-024-03942-5)
Supplement: Supplementary file 1 — Supplementary Information [file 41597_2024_3942_MOESM1_ESM.pdf]

Table of Contents

Supplementary Information	Title
Table S1	Individual weights and hormone levels of mice
Figure S1	RNA quality of each adipose tissue sample
Figure S2	Sequencing quality score plots

Table S1

Sample	Weight (g)	Estrogen (ng/mL)	Progesterone (ng/mL)
proestrus-1	21.3	0.45	0.90
proestrus-2	22.7	0.46	1.52
proestrus-3	24.1	0.48	1.25
proestrus-4	20.9	0.44	1.82
proestrus-5	23.4	0.45	2.44
estrus-1	21.8	0.37	7.75
estrus-2	22.3	0.37	7.49
estrus-3	24.5	0.10	5.48
estrus-4	20.6	0.34	4.22
estrus-5	23.9	0.35	9.80
metestrus-1	24.2	0.28	35.96
metestrus-2	21.5	0.55	33.04
metestrus-3	22.9	0.58	26.63
metestrus-4	20.8	0.40	28.72
metestrus-5	23.1	0.51	13.71
diestrus-1	24.3	0.18	4.34
diestrus-2	21.2	0.18	7.28
diestrus-3	22.6	0.25	9.03
diestrus-4	23.8	0.29	7.60
diestrus-5	22.1	0.46	11.48

Table S1. Individual weights and hormone levels of mice.

Figure S1

a **b**

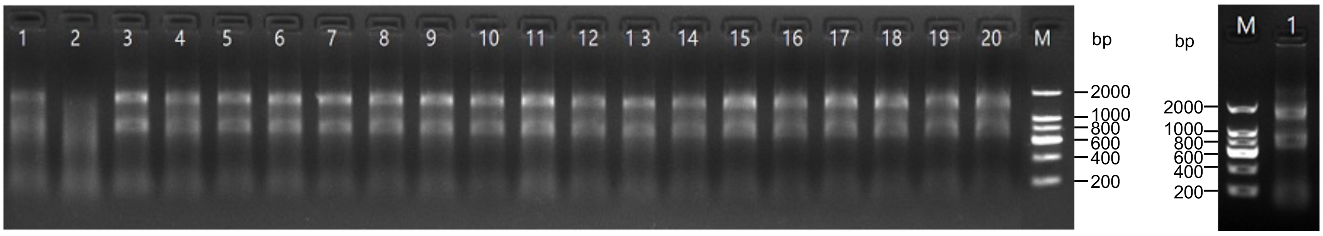

c

Sample	OD260/280	RNA concentration (ng/μL)
proestrus-1	1.88	125.0
proestrus-2	1.95	332.0
proestrus-3	1.93	310.0
proestrus-4	2.10	262.0
proestrus-5	1.87	141.0
estrus-1	2.03	93.2
estrus-2	1.98	60.6
estrus-3	1.91	262.0
estrus-4	2.12	94.0
estrus-5	1.85	77.8
metestrus-1	1.97	199.0
metestrus-2	2.06	127.0
metestrus-3	2.14	53.8
metestrus-4	1.92	115.0
metestrus-5	2.02	185.0
diestrus-1	1.89	127.0
diestrus-2	2.07	193.0
diestrus-3	2.01	152.0
diestrus-4	1.94	200.0
diestrus-5	2.08	214.0

Figure S1. RNA quality of each adipose tissue sample. a. Agarose gel electrophoresis images for 20 adipose tissue samples. Samples 1 to 5 correspond to proestrus-1 to proestrus-5, samples 6 to 10 correspond to estrus-1 to estrus-5, samples 11 to 15 correspond to metestrus-1 to metestrus-5, and samples 16 to 20 correspond to diestrus-1 to diestrus-5. The RNA quality of the adipose tissue from proestrus-2 was insufficient for further experiments. Therefore, we used another portion of adipose tissue from the same animal, which had been stored at -80°C, and its agarose gel electrophoresis image is shown in b. c. OD260/280 ratio and RNA concentration for each sample.

Figure S2

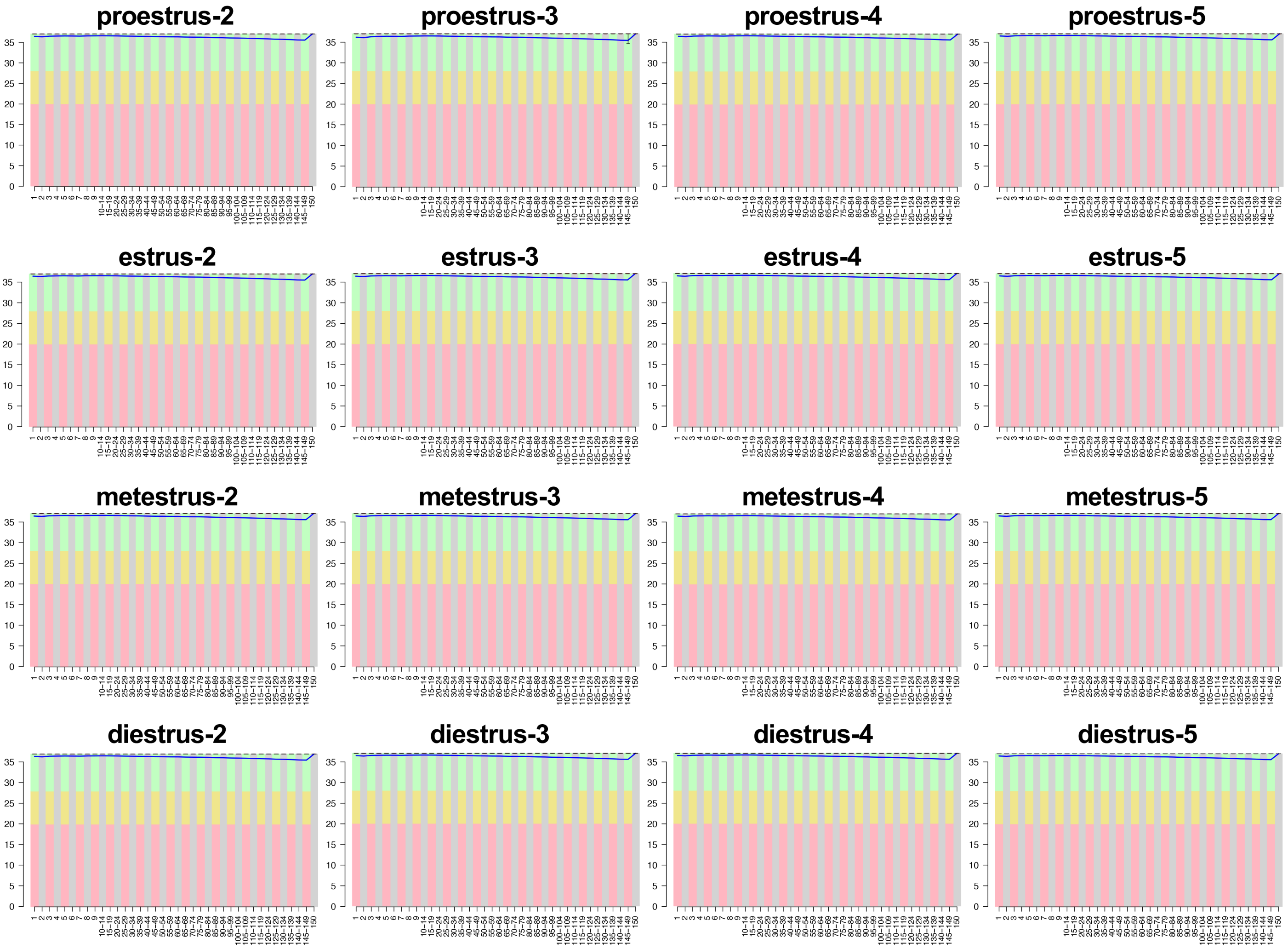

Figure S2. Sequencing quality score plots. The x-axis represents the base position in the sequence, and the y-axis represents the quality scores.
